# Biodiversity and socioeconomics in the city: a review of the luxury effect

**DOI:** 10.1098/rsbl.2018.0082

**Published:** 2018-05-09

**Authors:** Misha Leong, Robert R. Dunn, Michelle D. Trautwein

**Affiliations:** 1 Institute for Biodiversity Science and Sustainability, California Academy of Sciences, San Francisco, CA, USA; 2 Department of Applied Ecology and Keck Center for Behavioral Biology, North Carolina State University, Raleigh, NC, USA; 3 Center for Macroecology, Evolution and Climate, Natural History Museum of Denmark, University of Copenhagen, Denmark; 4 The German Centre for Integrative Biodiversity Research (iDiv), Liepzig, Germany

**Keywords:** luxury effect, biodiversity, socioeconomics, urban ecology

## Abstract

The ecological dynamics of cities are influenced not only by geophysical and biological factors, but also by aspects of human society. In cities around the world, a pattern of higher biodiversity in affluent neighbourhoods has been termed ‘the luxury effect'. The luxury effect has been found globally regarding plant diversity and canopy or vegetative cover. Fewer studies have considered the luxury effect and animals, yet it has been recognized in the distributions of birds, bats, lizards and indoor arthropods. Higher socioeconomic status correlates with higher biodiversity resulting from many interacting factors—the creation and maintenance of green space on private and public lands, the tendency of both humans and other species to favour environmentally desirable areas, while avoiding environmental burdens, as well as enduring legacy effects. The luxury effect is amplified in arid cities and as neighbourhoods age, and reduced in tropical areas. Where the luxury effect exists, benefits of urban biodiversity are unequally distributed, particularly in low-income neighbourhoods with higher minority populations. The equal distribution of biodiversity in cities, and thus the elimination of the luxury effect, is a worthy societal goal.

## 1. Introduction

Most of the history of ecology has focused on aspects of climate and geography as determinants of the abundance, composition and diversity of species found in a place. However, the social systems of humans also influence biodiversity. Even in the earliest cities, socioeconomics had an impact on species distributions and biodiversity. For example, in 1350 BCE Amarna, Egypt, the living areas of labourers hosted different insect species compared to those in affluent neighbourhoods [1]. Similarly, in early public health research in nineteenth century London, Snow found that the distribution of cholera was related to the distance of people from a contaminated well; however, an alternate hypothesis related to a connection between poverty and cholera [2]. More recently, Hope and colleagues [3], identified a curious pattern in Phoenix, Arizona, which they termed the luxury effect, wherein more affluent neighbourhoods have higher plant diversity. Since then, scientists have recognized the luxury effect in cities around the world, exploring various urban ecosystems and measures of plant and animal biodiversity.

## 2. Where past research has found the luxury effect

When Hope *et al.* [3] first proposed the luxury effect, they considered patterns of plant diversity in the Central Arizona-Phoenix long-term ecological research study area. They sampled all perennial, woody plants in 30 by 30 m plots and then tested the correlation between their diversity and geophysical, geographical and social variables. Along with elevation and land-use history, they discovered that census block median household income was one of the most important predictor variables [3]. Wealthier census blocks tended to have a higher diversity of woody plants. Hope and colleagues sampled both green and non-green parts of the city, so the relationship between biodiversity and affluence they observed included both the effects of affluence on greenness (whether a plot had plants at all) and then for those plots with plants, the number of species present.

The luxury effect has since been found in respect to plant diversity in North America, Burundi, China and Australia [4–9]. Other studies have used the concept of the luxury effect to describe the effect of affluence, not on plant diversity per se, but on the greenness of neighbourhoods in cities, measured using remote sensing tools focused on tree canopy or ground vegetation cover [10–15]. Greenness of cities, particularly canopy cover, sets the template for patterns in the composition and diversity of other species. Socioeconomics has been shown, in some cases, to outperform other biophysical and human factors in predicting plant diversity or vegetation cover [10,14,16]. A caveat to this trend is that we suspect that absence of the luxury effect is underreported. If socioeconomics is not an important variable, its lack of importance is unlikely to be featured.

Humans act directly on urban vegetation, from selecting plants to introduce and maintain to actively eliminating others. Our influence over more mobile taxa is indirect, yet the luxury effect can still be observed in animals, demonstrating the wide reach of socioeconomics. Fewer studies have considered the effects of socioeconomics on animals than on plants, and most of the animal studies have considered birds. Positive correlations between neighbourhood income and bird diversity are common and have been reported in North America, Europe and New Zealand [17–22].

Bird community composition can also be correlated with socioeconomics. Kinzig *et al.* [17] found that, in Phoenix, the greater diversity of birds in parks in high-income neighbourhoods than parks in low-income neighbourhoods was driven by differences in the number of native bird species. Lerman & Warren [19] also found that the abundance of native, desert bird species was highest in higher-income neighbourhoods in Phoenix. Similarly, Melles [18] found that the diversity of native bird species increased with socioeconomic status in Vancouver, Canada. The effects of socioeconomics on the proportion of native and introduced bird species do not, however, seem general. While Loss *et al.* [20] found that bird diversity was, indeed, higher in more affluent areas of Chicago, this effect was driven by introduced bird species. Native bird diversity was actually greater in low income neighbourhoods. No general expectation for when affluence might favour native versus introduced biodiversity has emerged, though the Phoenix studies suggest that a template of native plant diversity influences the presence of native bird species [17,19].

Birds depend directly on plant species for food and nesting, so it is possible that their diversity correlating with socioeconomics is, in part, mediated by the effect of affluence on plants. However, animals that are less reliant on vegetation have also exhibited luxury effect patterns, implying that other factors of affluence, independent of vegetation effects, may also influence ecological communities. To date, the biodiversity of both lizards and bats appears to be correlated with affluence in cities. One study, again in Phoenix, by Ackely *et al.* [23] found increased lizard diversity in affluent areas and that socioeconomic variables proved to be better predictors of diversity than other environmental variables. In Texas, Li & Wilkins [24] found that certain bat species were more often found in high-income neighbourhoods, while other bat species did not demonstrate any relationship with socioeconomics. A study of opossums, coyotes and raccoons in Chicago focused on the abundance or occupancy of individual species rather than overall diversity [25]. Results differed between taxa, but showed that socioeconomic factors performed just as well as the tested environmental factors in modelling distributions [25].

Only one study has considered the effect of affluence on arthropods, despite their diversity and abundance in cities. Leong and colleagues [26] found that homes in wealthier census blocks in North Carolina were more likely to have higher arthropod diversity indoors, regardless of vegetation at the property level. Further, while millions of species of microbes live in cities [27], no studies have considered how affluence might affect their biodiversity. Studies have, however, shown that urban residents of low socioeconomic status have more inflammatory and immune diseases—possibly due to their lack of exposure to microbial diversity that exists in green spaces, animals and agricultural settings [28].

Most of the studies of the luxury effect focus on terrestrial environments, but a few consider aquatic environments. So far, organisms in aquatic environments do not appear to exhibit a luxury effect. In one study, the biodiversity of pond macroinvertebrates around Stockholm, Sweden was unaffected by socioeconomic variables. A study from England actually found that ponds in affluent neighbourhoods have fewer sensitive aquatic invertebrate taxa [29]. The species missing from more affluent areas tended to be those less tolerant of pollutants, leading the authors to hypothesize that more two-car households in affluent neighbourhoods leads to more toxic run-off into nearby ponds [29].

## 3. Measuring the luxury effect

The luxury effect describes a relationship between ‘wealth' and ‘biodiversity,' but there are many ways of measuring these features. Wealth is often measured as a function of median household income, but upon proposing the luxury effect, Hope *et al.* [3] hypothesized the association between household income and biodiversity could also be due to correlating socioeconomic factors such as education, cultural values and institutional power. Future work, teasing apart the aspects of wealth and socioeconomics that matter most will be important, but in practice, median household income seems to be both a strong correlate of biodiversity and a proxy for many other socioeconomic features.

As with socioeconomics, techniques for evaluating ‘biodiversity' also vary. Many use α diversity, or richness [3–8,17–20,23,26], whether measured at a single square metre [30] or whole city blocks. Other measurements of diversity that require abundance data, such as community composition, diversity indices, density or evenness are less frequently used [17,20,31]. A few studies of focal taxa, such as bats or other urban mammals, where α diversity is less informative, tend to focus on the abundance or occupancy of individual species to model how these groups are distributed throughout cities. Few, if any, studies appear to have considered whether the among site (β) diversity or total (γ) diversity of less affluent neighbourhoods differs from that of more affluent neighbourhoods.

Studies of the luxury effect use the term ‘biodiversity' in a broad sense, and include vegetation studies that do not measure plant diversity on the ground, but instead use remote sensing tools to evaluate canopy and vegetation ground cover (10–15), given the assumption that these indirect measurements still provide information on biodiversity patterns [32]. There is no reason to expect the patterns of ‘biodiversity' and affluence to be the same for these disparate metrics of biodiversity. It would be useful to compare the affluence effect for one group of organisms at different sampling grains and with each of many metrics of biodiversity.

## 4. Factors driving the luxury effect

What explains the relationship between socioeconomics and urban biodiversity where it does occur? Here we argue that the primary links between biodiversity and affluence in cities relate to the area of green space (or canopy cover) which is influenced both by the decisions of individual property owners and city officials. Wealthier individuals and municipalities simply have more resources to allot towards vegetation and habitat. Vegetation in the urban landscape has often been introduced, and in many cases maintained, by human residents. The resultant novel plant community then has cascading effects on the surrounding ecological community. Additional factors of affluence beyond vegetation also contribute to luxury effect patterns.

### (a) Private properties

In many cities, private green spaces, including gardens, make up a relatively large proportion of total urban green space [33]. How residents choose to manage their land is influenced by disposable income, culture and individual values [34–36]. The first of these management choices is whether to leave a patch of ground as green/open space. In Hope *et al*. [3], for example, many plots sampled in lower income neighbourhoods had woody plant biodiversity of zero. Then, if individuals choose to maintain green spaces, they make choices about the identity and origin of the species they plant. While high-income neighbourhoods often have greater plant diversity than low-income neighbourhoods, ornamental plants (which are often exotic) can be favoured such that there is an inverse luxury effect for native plants [37]. Or, in the case where monoculture lawns are favoured, the influence of affluence on biodiversity may actually be negative. Additionally, what is desirable within a society varies—the racial/ethnic make-up of a neighbourhood, in addition to household income, was found to influence the composition of plants in community gardens in LA County [38]. Links between behaviour, culture and biodiversity, provide a mechanism to intentionally shift biodiversity patterns in cities in favour of those beneficial to conservation or ecosystem services to humans [39].

Decisions made at the property level can also impact animal communities. Besides the fact that property-level vegetation provides food, shelter and building materials for animals, human behaviour may also directly influence animal distributions. People around the world engage in wildlife feeding, and the abundance of households that feed birds increases in neighbourhoods of higher socioeconomic status [40]. A higher density of feeding stations is then related to greater abundance of visiting birds [40].

### (b) Public spaces

Public spaces, such as parks, medians and public right-of ways, account for much of the green space in urban areas. Socioeconomics affects the creation and maintenance of public green spaces through property taxes and through the effect of affluence on decision makers who determine where green spaces will be placed and maintained. As such, public green spaces are often inequitably distributed [14,41], as are street trees [8]. There is generally less urban tree canopy cover in neighbourhoods with lower income, lower education and more people of colour [14,42,43].

Well-documented environmental equity issues exist in urban environments such that low-income neighbourhoods (and those with higher racial/ethnic minority populations) shoulder greater public environmental burdens, such as exposure to pollutants [44]. This impacts not only human residents, but also other animals. For example, grey squirrels living in low-income neighbourhoods were found to have higher lead concentrations in their kidneys than those from more affluent neighbourhoods [45].

Considering the influence of affluence on the distribution of public green spaces, we predict that the luxury effect may be reduced in more socialist cities, where income inequality is reduced, and urban planning is more focused on the equitable distribution of city resources. In the USA then, where income inequality is among the most extreme in the developed world, the prevalence of the luxury effect may be expected.

### (c) Neighbourhood choice

Biodiversity might also be greater in affluent neighbourhoods because people of means may preferentially choose to live in habitats that naturally favour biodiversity [46,47]. Property values may then be higher in biodiverse neighbourhoods due to the increased competition to live there. For example, higher elevation sites in Phoenix are more biodiverse and have higher property values due, in part, to the aesthetics of biodiversity and/or of the conditions associated with it. In addition to being able to choose the best present-day neighbourhoods, affluent individuals have been able to choose the historically best parts of the city to develop their neighbourhoods whether that be near bodies of water, in better microclimates, or on hillsides [46,47].

### (d) Legacy effects

Especially in older cities, relationships between socioeconomics and biodiversity may be influenced not only by current patterns in green space and affluence, but also by legacies of land use [48]. Land-use history influences the distribution and composition of species in forests and other ecosystems [49], and may be particularly important in urban landscapes. In some cases, legacy effects modulate which species are influenced by affluence. For example, legacy effects may be larger on species with long generation times (e.g. trees), whereas species with shorter generation times (annuals) might respond more to current patterns in affluence in land use [41]. It generally appears that the luxury effect is amplified as neighbourhoods age because of the accumulation of established vegetation and the cascading ecological effects [3,9,16].

Social histories of cities also matter, and may explain deviations from expected patterns. While there is generally less canopy cover in neighbourhoods with more people of colour [14,42], regions exhibit contrasting patterns. For example, in the city centre of Detroit, Michigan, remote-sensing data showed that areas with the most increased vegetation over time were associated with the lowest household income [50]. This study, spanning decades, captured this inverse relationship that reflected a history of wealthier residents abandoning the urban centre and migrating to suburbs during the desegregation era.

### (e) Environmental context and regional differences

Vegetation luxury effect patterns have been found around the world [3,6,10,11,14,15], yet the strength of the luxury effect is, in part, contingent on the background ecosystem in which a city is built. For example*,* although urbanization has a tendency to decouple vegetation from precipitation compared to neighbouring wildland areas [15] a particularly strong relationship between affluence and biodiversity has been found in water-limited regions [14,15]. The earliest research on the luxury effect took place in the arid southwestern USA, which is extremely water limited. In arid cities, one of the direct mechanisms by which residents influence biodiversity is through water. In cities with limited vegetation and greater urban heat effects, increased water may lead to increased biodiversity, including potential influx of warm-adapted species [51], setting the stage for potentially complex affluence–biodiversity relationships. As arid regions are predicted to become drier with climate change [52], we predict that luxury effects are likely to become more pronounced in cities located in these zones in the future.

Few studies of the luxury effect have been done in the tropics. However, one study from tropical Bujumbura, Burundi found a positive relationship between affluence and plant diversity [4]. By contrast, a study in Puerto Rico did not document a relationship between household income and plant diversity [53]. Melendez-Ackerman *et al.* [53] hypothesize that the lack of a luxury effect in this case may be because water (and temperature) is not a limiting factor and that the income gap between neighbourhoods is less exaggerated than in other studies where the luxury effect has been supported.

### (f) Correlative nature of proposed mechanisms

The mechanisms through which affluence affects green space and biodiversity described here are non-exclusive and correlative. Affluent areas often have bigger private lots, more public green spaces and are, simultaneously, located in areas with high historic biodiversity. These different effects of affluence on biodiversity are then amplified with feedback loops: neighbourhoods are more desirable if they are closer to green spaces and have greater canopy cover, leading to higher property values, reinforcing an influx of high-income residents. Teasing apart the mechanisms driving the luxury effect and the associated correlational factors is an important next step.

## 5. Future directions

Urban affluence effects on biodiversity are common but not ubiquitous. Clearly one future direction is to better understand when they do and do not occur. Comparative studies between varying cities could disentangle the role of correlated factors in driving the luxury effect. For example, population density has a mixed association with the luxury effect [3,54]. Comparing the luxury effect in cities where the wealthiest neighbourhoods are in less population-dense suburbs, to cities where affluent neighbourhoods are in the city centres (ex. USA versus Europe) [55] could elucidate the relationship between population density and the luxury effect. Existing studies that compare cities have focused exclusively on canopy cover [14,15] measured with remote sensing tools. Canopy cover data alone leave unanswered questions about the specifics of plant and animal diversity. Future ground-truthing of canopy cover studies may come from citizen science data collection [56], or even from large-scale efforts at digitizing natural history collections [57].

Another, perhaps more important, future direction is working to better understand the cascading consequences of luxury effect patterns on daily human life. Many studies consider the consequences of biodiversity on human health and on ecological processes. Yet this literature, grounded in the study of wild places, offers many hypotheses yet to be tested in light of biodiversity patterns in cities. Better understanding the ecological processes behind the luxury effect will allow us to make predictions about the resiliency of urban ecosystems. For example: do affluence effects result in top down effects of predators and parasitoids on pests? Are more diverse neighbourhoods more resilient to the arrival of novel pests and pathogens? Overall, are they more resistant to change? The opportunities for new insights are great.

## 6. Broader implications

Increasingly, the ecosystem services provided by biological diversity are recognized [58]. In cities, these benefits include air and water purification, local climate moderation, CO_2_ sequestration, reduction in soil erosion and alleviation of noise pollution. Higher biodiversity is also associated with increased human psychological well-being, stronger social cohesion and community empowerment, and decreased crime. More recently, it has become clear that biodiversity also has beneficial effects on immune health. Higher biodiversity in gardens, for example, is associated with higher biodiversity on the skin of teens and, in turn, reduced allergy and other autoimmune problems [59]. The effects of biodiversity can, of course, also be negative [60] but they generally seem more likely to be positive, particularly at local scales. The luxury effect represents not just an interesting ecological pattern but also yet another layer in the social and structural injustices present in cities. The goal in studying luxury effects should be, in part, to figure out how to get rid of it. Our current understanding suggests that a healthy, ecologically sound city is one in which biodiversity is high and equitably distributed.

## Data accessibility

This article has no additional data.

## References

[1] Panagiotakopulu E 2001 New records for ancient pests: archaeoentomology in Egypt. J. Archaeol. Sci. 28, 1235–1246. (10.1006/jasc.2001.0697)

[2] Smith GD 2002 Commentary: behind the broad street pump: aetiology, epidemiology and prevention of cholera in mid-19th century Britain. Int. J. Epidemiol. 31, 920–932. (10.1093/ije/31.5.920) 12435761

[3] Hope D, Gries C, Zhu WX, Fagan WF, Redman CL, Grimm NB, Nelson AL, Martin C, Kinzig A 2003 Socioeconomics drive urban plant diversity. Proc. Natl Acad. Sci. USA 100, 8788–8792. (10.1073/pnas.1537557100) 12847293 PMC166391

[4] Bigirimana J, Bogaert J, De Cannière C, Bigendako M-J, Parmentier I 2012 Domestic garden plant diversity in Bujumbura, Burundi: role of the socio-economical status of the neighborhood and alien species invasion risk. Landsc. Urban Plan. 107, 118–126. (10.1016/j.landurbplan.2012.05.008)

[5] Clarke LW, Jenerette GD, Davila A 2013 The luxury of vegetation and the legacy of tree biodiversity in Los Angeles, CA. Landsc. Urban Plan. 116, 48–59. (10.1016/j.landurbplan.2013.04.006)

[6] Kirkpatrick JB, Daniels GD, Davison A 2011 Temporal and spatial variation in garden and street trees in six eastern Australian cities. Landsc. Urban Plan. 101, 244–252. (10.1016/j.landurbplan.2011.02.029)

[7] Wang H-F, Qureshi S, Knapp S, Friedman CR, Hubacek K 2015 A basic assessment of residential plant diversity and its ecosystem services and disservices in Beijing, China. Appl. Geogr. 64, 121–131. (10.1016/j.apgeog.2015.08.006)

[8] Avolio M, Pataki DE, Gillespie T, Jenerette GD, McCarthy HR, Pincetl S, Weller-Clarke L 2015 Tree diversity in southern California's urban forest: the interacting roles of social and environmental variables. Front. Ecol. Evol. 3, 73 (10.3389/fevo.2015.00073)

[9] Martin CA, Warren PS, Kinzig AP 2004 Neighborhood socioeconomic status is a useful predictor of perennial landscape vegetation in residential neighborhoods and embedded small parks of Phoenix, AZ. Landsc. Urban Plan. 69, 355–368. (10.1016/j.landurbplan.2003.10.034)

[10] Grove JM, Troy AR, O'Neil-Dunne JPM, Burch WR Jr, Cadenasso ML, Pickett STA 2006 Characterization of households and its implications for the vegetation of urban ecosystems. Ecosystems 9, 578–597. (10.1007/s10021-006-0116-z)

[11] Mennis J 2006 Socioeconomic–vegetation relationships in urban, residential land. Photogramm. Eng. Remote Sens. 72, 911–921. (10.14358/PERS.72.8.911)

[12] Lowry JH, Baker ME, Ramsey RD 2012 Determinants of urban tree canopy in residential neighborhoods: household characteristics, urban form, and the geophysical landscape. Urban Ecosyst. 15, 247–266. (10.1007/s11252-011-0185-4)

[13] Gong C, Yu S, Joesting H, Chen J 2013 Determining socioeconomic drivers of urban forest fragmentation with historical remote sensing images. Landsc. Urban Plan. 117, 57–65. (10.1016/j.landurbplan.2013.04.009)

[14] Schwarz K et al. 2015 Trees grow on money: urban tree canopy cover and environmental justice. PLoS ONE 10, e0122051 (10.1371/journal.pone.0122051) 25830303 PMC4382324

[15] Jenerette GD, Miller G, Buyantuev A, Pataki DE, Gillespie TW, Pincetl S 2013 Urban vegetation and income segregation in drylands: a synthesis of seven metropolitan regions in the southwestern United States. Environ. Res. Lett. 8, 044001 (10.1088/1748-9326/8/4/044001)

[16] Luck GW, Smallbone LT, O'Brien R 2009 Socio-economics and vegetation change in urban ecosystems: patterns in space and time. Ecosystems 12, 604 (10.1007/s10021-009-9244-6)

[17] Kinzig AP, Warren P, Martin C, Hope D, Katti M 2005 The effects of human socioeconomic status and cultural characteristics on urban patterns of biodiversity. Ecol. Soc. 10, 23 (10.5751/ES-01264-100123)

[18] Melles SJ 2015 Urban bird diversity as an indicator of human social diversity and economic inequality in Vancouver, British Columbia. Urban Habitats 3, 25–48.

[19] Lerman SB, Warren PS 2011 The conservation value of residential yards: linking birds and people. Ecol. Appl. 21, 1327–1339. (10.1890/10-0423.1) 21774433

[20] Loss SR, Ruiz MO, Brawn JD 2009 Relationships between avian diversity, neighborhood age, income, and environmental characteristics of an urban landscape. Biol. Conserv. 142, 2578–2585. (10.1016/j.biocon.2009.06.004)

[21] Strohbach M, Haase D, Kabisch N 2009 Birds and the city: urban biodiversity, land use, and socioeconomics. Ecol. Soc. 14, 31 (10.5751/ES-03141-140231)

[22] van Heezik Y, Freeman C, Porter S, Dickinson KJM 2013 Garden size, householder knowledge, and socio-economic status influence plant and bird diversity at the scale of individual gardens. Ecosystems 16, 1442–1454. (10.1007/s10021-013-9694-8)

[23] Ackley JW, Wu J, Angilletta MJ Jr, Myint SW, Sullivan B 2015 Rich lizards: how affluence and land cover influence the diversity and abundance of desert reptiles persisting in an urban landscape. Biol. Conserv. 182, 87–92. (10.1016/j.biocon.2014.11.009)

[24] Li H, Wilkins KT 2014 Patch or mosaic: bat activity responds to fine-scale urban heterogeneity in a medium-sized city in the United States. Urban Ecosyst. 17, 1013–1031. (10.1007/s11252-014-0369-9)

[25] Magle SB, Lehrer EW, Fidino M 2016 Urban mesopredator distribution: examining the relative effects of landscape and socioeconomic factors. Anim. Conserv. 19, 163–175. (10.1111/acv.12231)

[26] Leong M, Bertone MA, Bayless KM, Dunn RR, Trautwein MD 2016 Exoskeletons and economics: indoor arthropod diversity increases in affluent neighbourhoods. Biol. Lett. 12, 20160322 (10.1098/rsbl.2016.0322) 27484644 PMC5014024

[27] Kembel SW, Jones E, Kline J, Northcutt D, Stenson J, Womack AM, Bohannan BJ, Brown GZ, Green JL 2012 Architectural design influences the diversity and structure of the built environment microbiome. ISME J. 6, 1469–1479. (10.1038/ismej.2011.211) 22278670 PMC3400407

[28] Rook GAW, Raison CL, Lowry CA 2014 Microbial ‘old friends’, immunoregulation and socioeconomic status. Clin. Exp. Immunol. 177, 1–12. (10.1111/cei.12269) 24401109 PMC4089149

[29] Gledhill DG, James P 2012 Socio-economic variables as indicators of pond conservation value in an urban landscape. Urban Ecosyst. 15, 849–861. (10.1007/s11252-012-0242-7)

[30] Reese AT, Savage A, Youngsteadt E, McGuire KL, Koling A, Watkins O, Frank SD, Dunn RR 2016 Urban stress is associated with variation in microbial species composition—but not richness—in Manhattan. ISME J. 10, 751–760. (10.1038/ismej.2015.152) 26394011 PMC4817683

[31] Blicharska M, Andersson J, Bergsten J, Bjelke U, Hilding-Rydevik T, Thomsson M, Östh J, Johansson F 2017 Is there a relationship between socio-economic factors and biodiversity in urban ponds? A study in the city of Stockholm. Urban Ecosyst. 20, 1209–1220. (10.1007/s11252-017-0673-2)

[32] Turner W, Spector S, Gardiner N, Fladeland M, Sterling E, Steininger M 2003 Remote sensing for biodiversity science and conservation. Trends Ecol. Evol. 18, 306–314. (10.1016/S0169-5347(03)00070-3)

[33] Goddard MA, Dougill AJ, Benton TG 2010 Scaling up from gardens: biodiversity conservation in urban environments. Trends Ecol. Evol. 25, 90–98. (10.1016/j.tree.2009.07.016) 19758724

[34] Grove JM, Locke DH, O'Neil-Dunne JPM 2014 An ecology of prestige in new york city: examining the relationships among population density, socio-economic status, group identity, and residential canopy cover. Environ. Manage. 54, 402–419. (10.1007/s00267-014-0310-2) 25034751

[35] Goddard MA, Dougill AJ, Benton TG 2013 Why garden for wildlife? Social and ecological drivers, motivations and barriers for biodiversity management in residential landscapes. Ecol. Econ. 86, 258–273. (10.1016/j.ecolecon.2012.07.016)

[36] Belaire JA, Westphal LM, Minor ES 2016 Different social drivers, including perceptions of urban wildlife, explain the ecological resources in residential landscapes. Landsc. Ecol. 31, 401–413. (10.1007/s10980-015-0256-7)

[37] Davoren E, Siebert S, Cilliers S, du Toit MJ 2016 Influence of socioeconomic status on design of Batswana home gardens and associated plant diversity patterns in northern South Africa. Landsc. Ecol. Eng. 12, 129–139. (10.1007/s11355-015-0279-x)

[38] Clarke LW, Jenerette GD 2015 Biodiversity and direct ecosystem service regulation in the community gardens of Los Angeles, CA. Landsc. Ecol. 30, 637–653. (10.1007/s10980-014-0143-7)

[39] Larsen L, Harlan SL 2006 Desert dreamscapes: residential landscape preference and behavior. Landsc. Urban Plan. 78, 85–100. (10.1016/j.landurbplan.2005.06.002)

[40] Fuller RA, Warren PH, Armsworth PR, Barbosa O, Gaston KJ 2008 Garden bird feeding predicts the structure of urban avian assemblages. Divers. Distrib. 14, 131–137. (10.1111/j.1472-4642.2007.00439.x)

[41] Boone CG 2008 Environmental justice as process and new avenues for research. Environ. Justice 1, 149–154. (10.1089/env.2008.0530)

[42] Brooks KR, Kelley W, Amiri S 2016 Social equity of street trees in the pedestrian realm. Pap. Appl. Geogr. 2, 216–235. (10.1080/23754931.2015.1121163)

[43] Jesdale BM, Morello-Frosch R, Cushing L 2013 The racial/ethnic distribution of heat risk-related land cover in relation to residential segregation. Environ. Health Perspect. 121, 811–817. (10.1289/ehp.1205919) 23694846 PMC3701995

[44] O'Neill MS et al. 2003 Health, wealth, and air pollution: advancing theory and methods. Environ. Health Perspect. 111, 1861–1870. (10.1289/ehp.6334) 14644658 PMC1241758

[45] McKINNON JG, Hoff GL, Bigler WJ, Prather EC 1976 Heavy metal concentrations in kidneys of urban gray squirrels. J. Wildl. Dis. 12, 367–371. (10.7589/0090-3558-12.3.367) 16498879

[46] Harlan SL, Brazel AJ, Jenerette GD, Jones NS, Larsen L, Prashad L, Stefanov WL 2007 In the shade of affluence: the inequitable distribution of the urban heat island. In Equity and the environment (eds Wilkinson RC, Freudenburg WR), pp. 173–202. Bingley, UK: Emerald Group Publishing Limited.

[47] Meyer WB 1994 Bringing hypsography back in: altitude and residence in American Cities. Urban Geogr. 15, 505–513. (10.2747/0272-3638.15.6.505)

[48] Ramalho CE, Hobbs RJ 2012 Time for a change: dynamic urban ecology. Trends Ecol. Evol. 27, 179–188. (10.1016/j.tree.2011.10.008) 22093191

[49] Foster D, Swanson F, Aber J, Burke I, Brokaw N, Tilman D, Knapp A 2003 The importance of land-use legacies to ecology and conservation. BioScience 53, 77–88. (10.1641/0006-3568(2003)053%5B0077:TIOLUL%5D2.0.CO;2)

[50] Ryznar RM, Wagner TW 2001 Using remotely sensed imagery to detect urban change: viewing detroit from space. J. Am. Plann. Assoc. 67, 327–336. (10.1080/01944360108976239)

[51] Meineke EK, Dunn RR, Sexton JO, Frank SD 2013 Urban warming drives insect pest abundance on street trees. PLoS ONE 8, e59687 (10.1371/journal.pone.0059687) 23544087 PMC3609800

[52] Reager JT, Gardner AS, Famiglietti JS, Wiese DN, Eicker A, Lo M-H 2016 A decade of sea level rise slowed by climate-driven hydrology. Science 351, 699–703. (10.1126/science.aad8386) 26912856

[53] Meléndez-Ackerman E, Santiago-Bartolomei R, Vila-Ruiz C, Santiago L, García-Montiel D, Verdejo-Ortiz J, Manrique-Hernández H, Hernández-Calo E 2014 Socioeconomic drivers of yard sustainable practices in a tropical city. Ecol. Soc. 19, 20 (10.5751/ES-06563-190320)

[54] Locke DH, Grove JM 2016 Doing the hard work where it's easiest? Examining the relationships between urban greening programs and social and ecological characteristics. Appl. Spat. Anal. Policy 9, 77–96. (10.1007/s12061-014-9131-1)

[55] Brueckner JK, Thisse J-F, Zenou Y 1999 Why is central paris rich and downtown detroit poor? An amenity-based theory. Eur. Econ. Rev. 43, 91–107. (10.1016/S0014-2921(98)00019-1)

[56] Silvertown J 2009 A new dawn for citizen science. Trends Ecol. Evol. 24, 467–471. (10.1016/j.tree.2009.03.017) 19586682

[57] Vollmar A, Macklin JA, Ford L 2010 Natural history specimen digitization: challenges and concerns. Biodivers. Inform. 7, 93–112. (10.17161/bi.v7i2.3992)

[58] Sandifer PA, Sutton-Grier AE, Ward BP 2015 Exploring connections among nature, biodiversity, ecosystem services, and human health and well-being: opportunities to enhance health and biodiversity conservation. Ecosyst. Serv. 12, 1–15. (10.1016/j.ecoser.2014.12.007)

[59] Hanski I et al. 2012 Environmental biodiversity, human microbiota, and allergy are interrelated. Proc. Natl Acad. Sci. USA 109, 8334–8339. (10.1073/pnas.1205624109) 22566627 PMC3361383

[60] Dunn R 2010 Global mapping of ecosystem disservices: the unspoken reality that nature sometimes kills us. Biotropica 42, 555–557. (10.1111/j.1744-7429.2010.00698.x)

